# The FIFA 11 + Referees program improves knee proprioception in female futsal referees

**DOI:** 10.1038/s41598-025-20054-1

**Published:** 2025-09-17

**Authors:** Fatemeh Khosravi rad, Mostafa Zarei, Seyed Mohammad Hosseini, Mojtaba Asgari

**Affiliations:** 1 Faculty of sport sciences and health, Shahid Beheshti University, Tehran, Iran; 2 Movement and Training Science Group, Institute for Sport and Sport Science, TU Dortmund University, Dortmund, Germany

**Keywords:** FIFA + 11 referees, Injury risk, Knee joint proprioception, dynamic balance, Health care, Risk factors

## Abstract

While the FIFA 11 + Referees program (the 11 + Referees) is the only structured injury prevention program for referees, its effects on injury-related risk factors among referees remain underexplored. This study investigated the effects of the 11 + Referees on knee joint proprioception and dynamic balance. Thirty four female referees from the Tehran Premier Futsal League voluntarily participated and were randomly assigned to either a control group (*n* = 17) or the 11 + Referees group (*n* = 17). All participants underwent a baseline assessment including knee joint proprioception at angular velocities of 15°/s, 45°/s, and 60°/s, and position sense, as well as dynamic balance. Subsequently, the intervention group performed the 11 + Referees program three times weekly for 8 weeks, while the control group performed general warm-ups. Post-intervention, the same test battery was administered, and changes were analyzed to identify the program’s effects on the measures understudied. Significant improvements were observed in knee joint proprioception at 15°/s (F = 18.19, *p* < 0.001, η²=0.44), 45°/s (F = 4.07, *p* = 0.056, ηp²=0.15), and 60°/s (F = 6.63, *p* = 0.017, η²=0.22) in the intervention group. However, dynamic balance did not show significant improvement (F = 0.79, *p* = 0.38, η²=0.033). In conclusion, an eight-week application of the 11 + Referees enhanced knee joint proprioception in female referees with large effect sizes, but had no significant effect on dynamic balance. Improvements in proprioception may be attributed to neuromuscular adaptations resulting from regular program use. The absence of balance improvement could be due to the short intervention period and the hard futsal court surface, which may limit the effectiveness of certain balance exercises. Future studies should explore the program's effects on other injury risk factors and injury rates.

## Introduction

A 72% increase in qualified female referees between 2016 and 2020^1^ alongside a rapid global development of women’s football population within the past two decades^2^^3^ underscores a growing recognition of women’s roles in football. As essential figures in every football and futsal match, more than 840,000 referees (2018) across the world actively ensure fair play and match safety^4^. To maintain optimal positioning and make accurate decisions, referees must meet significant physical demands and should be in sync with the players^5^. Despite the increasing prominence of female players and referees, research dedicated to this population lags behind that of males across all disciplines^6^. Further, most football-related studies focus on the players, with limited research addressing referees, particularly female referees^7^.

The injury incidence among referees ranges from 5 to 34 injuries per 1000 h, a rate considered high^8^^9^. More than 40% of referees report at least one injury per season, with over 60% of complaints linked to musculoskeletal pain^6^. However, a review of the existing literature highlights a significant gap in prevention strategies aimed at safeguarding referees’ fitness and well-being^10^.

Futsal, a high-intensity sport, places substantial physical demands on referees, potentially increasing their risk of injury. Moreover, female futsal referees may face unique biomechanical and injury risks compared to players and male referees. This is primarily due to the repeated high-intensity actions, frequent stopping and pivoting on a hard indoor surface, and the need to maintain proximity to play while making quick decisions. These factors contribute to increased non-contact knee loading and may heighten the risk of ligament injuries, especially in female officials who generally exhibit greater joint laxity and neuromuscular differences compared to males. Despite this, research on fitness levels and match demands in female futsal referees remains scarce. Referees, unlike players, are primarily affected by non-contact injuries, which are both modifiable and preventable through structured exercise-based interventions^11^^12^. Given the consequences of injuries, the development and implementation of referee-specific injury prevention programs (IPPs) could offer substantial physical, financial, and socioeconomic benefits^13–17^.

The FIFA 11 + Referees program (the 11 + Referees) is the only structured exercise-based IPP specifically designed for referees. Unlike general injury prevention programs such as the standard FIFA 11 + or other warm-up protocols aimed primarily at players, the 11 + Referees was specifically developed to meet referees’ unique biomechanical and cognitive demands. This includes exercises that emphasize quick changes of direction, rapid acceleration and deceleration, and sustained focus on decision-making while moving, all of which is essential for maintaining optimal positioning and match control during play. Therefore, this program provides a more targeted approach for referees compared to generic warm-up routines^18^^19^. Developed based on referees’ unique demands, it is implemented as a warm-up routine aimed at enhancing muscle strength, neuromuscular control, and body awareness. The program consists of 18 exercises divided into three components: (1) running drills, (2) plyometric and balance exercises focusing on core strength, eccentric control, and proprioception, and (3) high-speed running with changes of direction^18^.

While the effectiveness of other FIFA 11 + programs (the original 11+, the 11 + Kids, the 11 + Shoulder) in reducing injury incidence and improving biomechanical and performance measures is well documented^20–24^research on the 11 + Referees remains limited^18^. In particular, its effects on injury risk factors such as knee joint proprioception and dynamic balance have yet to be explored. Given that female athletes are at a fourfold higher risk of knee injuries than their male counterparts, investigating the impact of the + 11 Referees program on these factors is crucial for optimizing its integration into female referees’ training routines. This study aims to address the effects of an eight-week the 11+ Referees program on knee joint proprioception and dynamic balance in female futsal referees. It is hypothesized that the intervention will lead to improvements in both proprioception and balance, thereby contributing to injury prevention efforts in this population.

## Materials and methods

### Etical considreations, registration and study design

This quasi-experimental study employed a repeated-measures design with an experimental and a control group and was registered at Iranian Registry of Clinical Trials: IRCT20240306061185N4, date:06/09/2024. The study adhered to the principles of the Declaration of Helsinki and the Data Protection Act 1998 and was approved by the Ethics Committee of Shahid Beheshti University (Ethical Code: IR.SSRC.REC.1402.069). Participation was voluntary and all participants received a comprehensive verbal and written explanation of the study protocol, and informed written consent was obtained before participation.

### Participants and procedures

Power analysis calculated a sample of 34 as suitable for an expected effect size of 0.5, an α error probability of 0.05 and a power of 0.80, aligning with previous research^27^. Hence, 34 referees from the Tehran Premier Futsal League (January-June 2023) were recruited and randomly assigned to either a control group (general warm-up, *n* = 17) or an intervention group (the 11 + Referees, *n* = 17), and underwent an 8-week intervention three times a week. At baseline, demographic characteristics (recorded using InBody co: BSM370 Stadiometer) and health status of the participants were documented. Following a familiarization session, participants completed a standardized test battery assessing knee joint proprioception and dynamic balance. Post-intervention, the same measurements were administered, and pre-to-post differences were analyzed to evaluate the training effects on the targeted outcomes. Data were collected in the Sports Injury and Corrective Exercises laboratory of Faculty of Sport Science and Health, Shahid Beheshti University, Tehran, Iran.

### Study criteria

Inclusion criteria were: (i) holding a second-class, first-class, or national referee certification from the Iranian Football Federation, (ii) officiating matches while also participating in three training sessions per week, and (iii) being injury-free at baseline. Exclusion criteria included: (i) a history of severe musculoskeletal disorders within three months before the study, (ii) missing any measurement sessions, and (iii) sustaining an injury during the study period.

### Training hours’ registration form

All training activities and officiating in official competitions from January to June 2023 were self-recorded by referees using a specialized form derived from the Comprehensive Statement of Football Injury Research^28^. Referees submitted their completed forms weekly to the researchers. Additionally, intervention group participants were required to document their use of the 11 + Referees program in each training session. To verify compliance, two research assistants conducted random visits to intervention group training sessions. Their observations were compared with the referees’ recorded reports to ensure the accuracy of self-reported program adherence (i.e., confirmation of 11 Referees program implementation).

### Test battery

Knee joint proprioception was measured using two methods: passive motion detection threshold and active joint position sensing at 15º/S, 45º/S, and 60º/S speeds using the Biodex isokinetic system pro 4 systems, manufactured in Switzerland by CMVAG Con-Trex.

### Active joint position sense

To assess joint position sense, participants actively moved their leg to a target angle (15°, 45°, or 60°) and maintained that position for five seconds before returning to the starting position (90°). They were then asked to replicate the target angle with their eyes closed to eliminate visual feedback and wearing headphones to prevent auditory cues. Each participant performed three trials per angle, and the mean absolute error was recorded. A 20-second rest period was provided between trials^29^.

### Passive movement detection threshold

The participant’s positioning and knee angle were set identically to the previous proprioception test. A handheld switch was provided to the participant, and the knee was passively moved into extension at a speed of 0.25°/s, starting from 0°. The participant was instructed to press the switch as soon as they detected any movement. To familiarize them with the procedure, two practice trials were conducted before the actual test. The test was then performed three times, and the average movement detection threshold was recorded. The same protocol was subsequently repeated for knee flexion^30^.

### Dynamic balance measurement

Dynamic balance was assessed using the Biodex Balance System SD (Biodex, USA) while standing on one leg at stability levels 5 to 8. The device allows for adjustments across 12 instability levels, ranging from nearly stable (level 12) to highly unstable (level 1). It consists of a circular platform mounted on a large sphere containing multiple sensors, enabling movement in various directions relative to the horizontal plane. The platform is highly sensitive to shifts in the center of gravity and responds to foot pressure changes by altering its tilt based on the applied torque. The system recorded deviations of the center of gravity from the base of support in real time, providing three stability indices: overall stability index, anterior-posterior stability index, and medial-lateral stability index. Participants were instructed to maintain balance at the center of the platform for 20 s while standing on one leg.

### Intervention

The intervention started at the beginning of the season and lasted for eight weeks. The control group engaged in a conventional warm-up routine, consisting of 5 min of low-intensity jogging followed by 10 min of static stretching exercises targeting major lower limb muscle groups (e.g., quadriceps, hamstrings, and calves). This warm-up approach mirrors the usual pre-match preparations practiced by the participants prior to the study. The 11 + Referees consists of three parts with 18 exercises, lasting 20–22 min per session in practice. The first part, which lasts eight minutes, includes six exercises designed to increase heart rate while emphasizing proper posture and alignment during running. The second part, lasting ten minutes, consists of eight exercises focusing on core and lower extremity stability, balance, and plyometric/agility training. This section has two difficulty levels, progressing every four to six weeks based on referee performance and adaptation. The third part, lasting two minutes, includes four high-speed running and jogging drills incorporating change-of-direction movements to enhance agility and match-specific conditioning (Table 1)^18^. To ensure accurate implementation and adherence to the intervention, two certified trainers supervised the execution of the 11 + Referees program during each session. They provided verbal cues and real-time feedback to correct movement techniques and ensure safety. Additionally, participants self-recorded their adherence using a standardized training log. Weekly spot checks were conducted by research staff to cross-verify self-reported data with actual performance and attendance.

**Table 1:** FIFA 11 + Referees Manual.

Row	No	Exercise Name	Sets × Reps/Duration	Biomechanical Goal
Part 1: slow running exercises				
R/AR	1	Running straight ahead	2	Warm-up, increase HR
	2	Running circling partner	2	Coordination, agility
	3	Running zigzag shuffling	2	Direction change, agility
	4	Running forwards and backwards sprints	2	Acceleration/deceleration
MR	5	Running slalom forwards and backwards	2	Agility, quick footwork
	6	Running forwards and backwards with rotations	2	Dynamic stability, coordination
AR	5	Running alternate shuffling	2	Lateral movement control
	6	Running cariocas	2	Hip mobility, coordination
**Part 1–2: Strength**,** Plyometric and Balance exercises**				
R/AR	1	The bench alternate legs	1 × 30–60 s	Core stability
	2	sideways bench raise and lower hip	1 × 20–30 s (each side)	Core, lateral stability
	3	The bridge alternate	1 × 20–30 s	Hip extensors, core
	4	Hamstrings basic	1 × 7–10 Reps.	Hamstring strength
	5	Calf basic	1 × 20–30 s (each side)	Calf strength
	6	Double leg squat jumps	1 × 20–30 s	Power, plyometric ability
MR	7	Single-leg Stance move the other leg	1 × 30 s (each side)	Balance, neuromuscular control
	8	Front lunges	1 × 10 Moves (each side)	Quadriceps, glute activation
AR	7	Lateral lunges 45°	1 × 10 Moves (each side)	Hip abductor strength
	8	lateral jumps	1 × 20–30 s	Plyometric power, coordination
**Part 2–2: Strength**,** Plyometric and Balance Exercises**				
R/AR	1	The bench one leg lift and hold	3 × 20–30 s (each side)	Advanced core control
	2	Sideways bench with leg lift	1 × 20–30 s (each side)	Core/lateral stability
	3	The bridge on one leg	1 × 20–30 s (each side)	Single-leg hip strength
	4	Hamstring advanced	1 × 10–15 Reps.	Hamstring strength
	5	Calf advanced	1 × 20–30 s	Calf endurance
	6	Double leg bounding	1 × 20–30 s	Power, plyometric skill
MR	7	Single leg cross hops	1 × 30 s (each leg)	Balance, agility
	8	scissor jumps	1 × 20–30 s	Plyometric explosiveness
AR	7	Lateral lunges 90°	1 × 10 Moves (each side)	Deep hip range, strength
	8	Double lateral jumps	1 × 20–30 s	Advanced plyometric
**Part 3: Fast Running Exercises**				
R/AR	1	Running progression run	2	Acceleration, endurance
	2	Running long sprints	2	Speed, stamina
MR	3	Running short diagonal sprint	2	Agility, speed
	4	Running long diagonal sprint	2	High-speed agility
AR	3	Running shuffling and short sprints	2	Quick reaction
	4	Running shuffling and long sprint	2	High-speed endurance

R– Referee, MR– Middle Referee, AR– Assistant Referee, Sec– seconds, Reps– repetitions.

### Statistical analysis

The normality of the data was assessed using the Shapiro-Wilk test, and homogeneity of variances was verified with Levene’s test. A paired sample t-test was used to compare pre-test and post-test scores within each group. To evaluate the effect of the 11 + Referees on knee joint proprioception and dynamic balance, an analysis of covariance (ANCOVA) was performed, with pre-test scores included as covariates. The significance level was set at *p* < 0.05, and statistical analyses were conducted using SPSS 29.

## Results

**Fig. 1:**
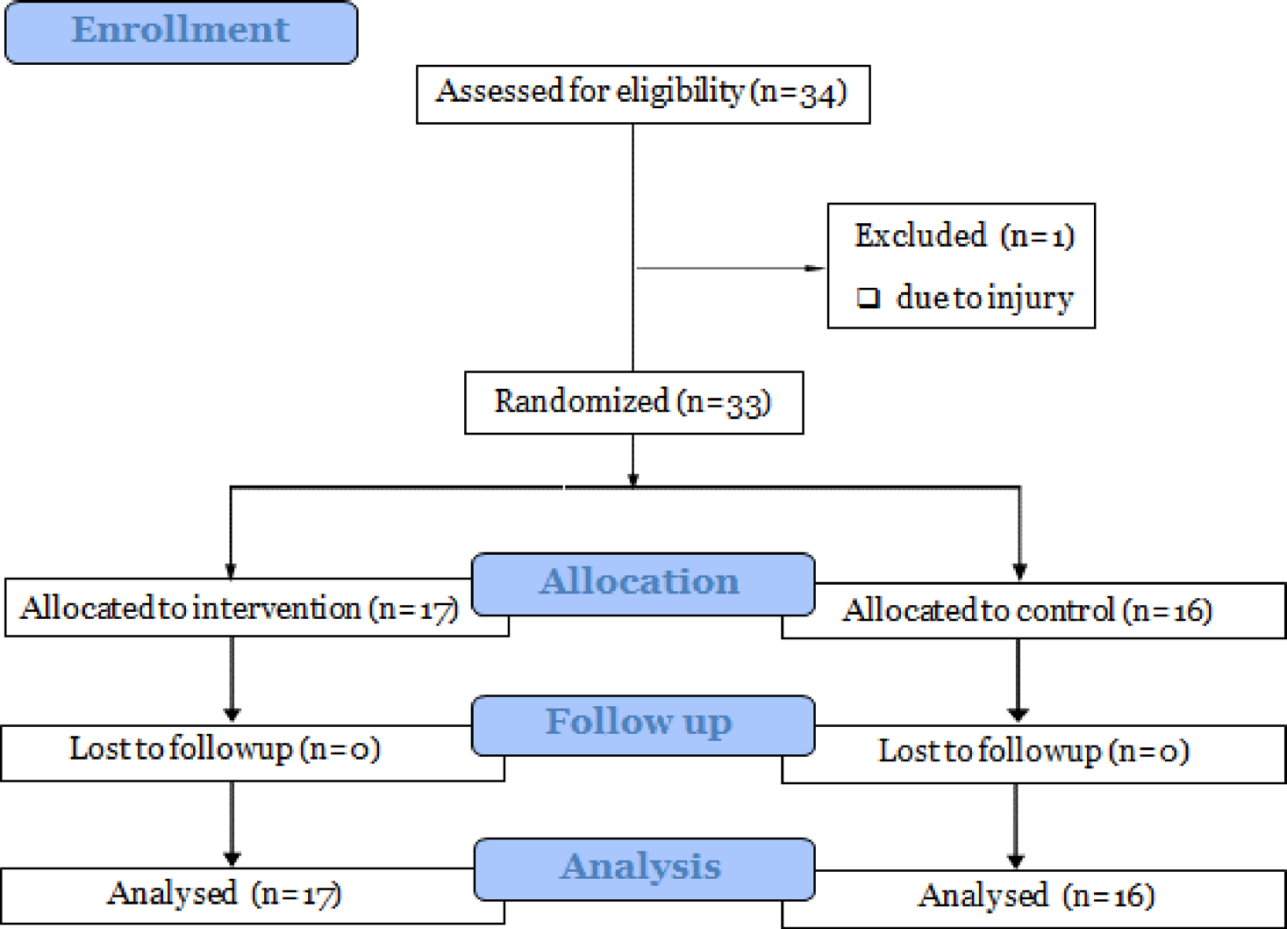
Study flowchart.

Out of the 34 participants, one withdrew from the study due to missing training sessions (Fig. 1). Consequently, data from 33 referees were analyzed, with 17 in the intervention group and 16 in the control group. Prior to testing, none of the participants reported physical complaints, and no time-loss injuries occurred in either group during the study period. All referees in the intervention group successfully progressed to level three of the training program. Additionally, there were no significant differences (*p* > 0.05) between the two groups in demographic variables such as age, height, weight, Body Mass Index (BMI), knee joint proprioception, and dynamic balance (Table 2).

**Table 2:** Descriptive of the variables and the results of the independent sample Test.

				Control group	Intervention group	T	df	Sig.
Variables				Means (SD)	Means (SD)			
Age				23.03 (7.05)	23.84 (6.13)	−0.35	31	0.72
Height				163.61 (5.26)	160.85 (4.91)	1.55	31	0.13
Weight				59.70 (11.04)	61.82 (5.48)	−0.70	31	0.48
Body Mass Index				22.24 (3.73)	24.01 (3.03)	−1.49	31	0.14
Knee Joint Proprioception	position sense	15°/s	Pre-test	2.53 (0.91)	4.01 (1.29)	−3.32	31	0.15
			Post-test	2.55 (1.04)	1.80 (1.01)	1.83	31	0.77
		45°/s	Pre-test	3.07 (1.39)	3.77 (1.52)	−1.22	31	0.91
			Post-test	2.18 (0.68)	1.65 (1.06)	1.47	31	0.04
		60°/s	Pre-test	3.02 (1.41)	3.20 (1.44)	−0.31	31	0.51
			Post-test	2.48 (1.27)	1.53 (1.04)	2.08	31	0.39
	Motion Sense		Pre-test	3.00 (1.38)	2.86 (1.15)	0.27	31	0.39
			Post-test	2.80 (1.44)	2.56 (1.38)	0.42	31	0.95
Dynamic Balance			Pre-test	1.18 (0.54)	1.79 (0.55)	−2.83	31	0.89
			Post-test	0.99 (0.44)	1.29 (0.50)	−1.61	31	0.44

SD – Standard Deviation, T – T statistic, df – degrees of freedom, Sig – Significance

### Effect of the 11+ Referees on knee joint proprioception of female futsal referees

**Fig. 2:**
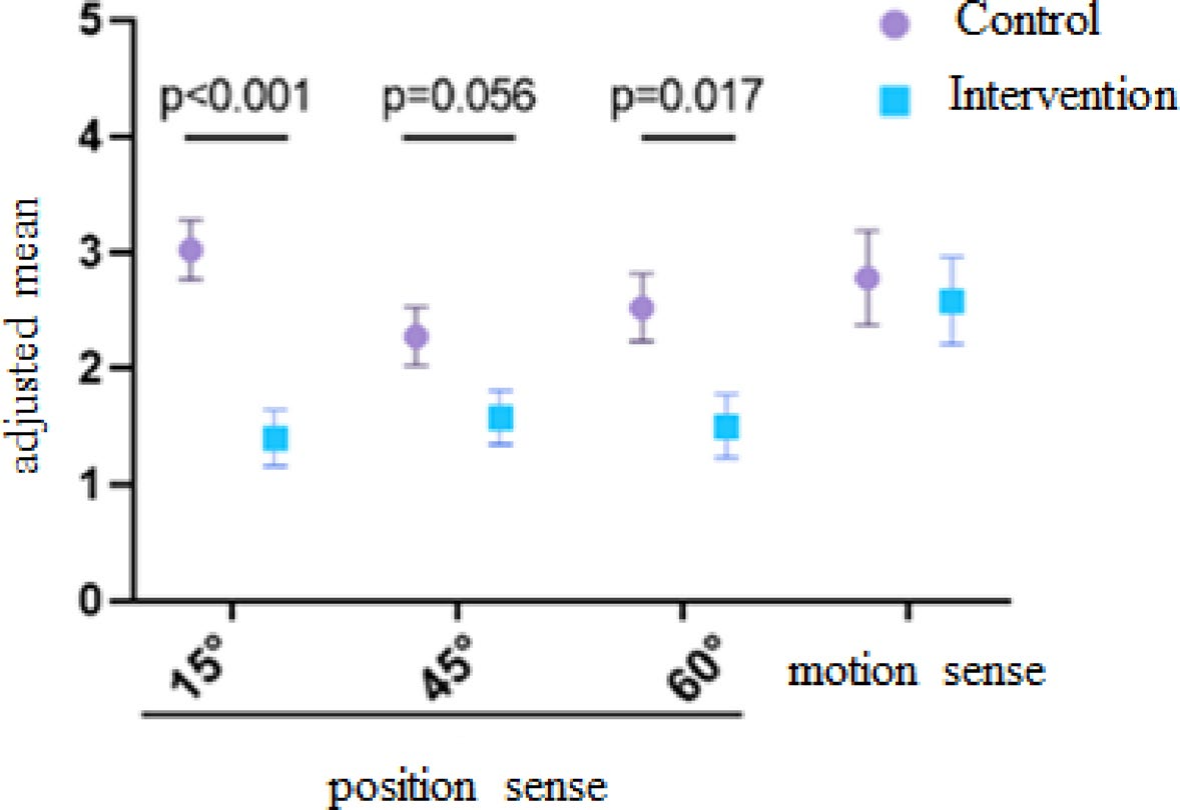
An adjusted mean difference of motion sense and position sense at speeds 15º/S, 45º/S, and 60º/S.

The ANCOVA results indicated a significant effect of the 11 + Referees program on knee joint proprioception at 15°/s (ηp² = 0.44, *p* < 0.001, F = 18.19), 45°/s (ηp² = 0.15, *p* = 0.056, F = 4.07), and 60°/s (ηp² = 0.22, *p* = 0.017, F = 6.63) when controlling for pre-test values. This marginal result at 45°/s may be due to the relatively small sample size, which could have reduced the statistical power for detecting a significant difference at this speed. According to Cohen’s thresholds (0.01 = small, 0.06 = medium, 0.14 = large), the effect sizes observed (ηp²) indicate large effects at 15°/s, moderate effects at 60°/s, and small to moderate effects at 45°/s. However, no significant differences were found between the groups in motion sense (ηp² = 0.006, *p* = 0.72, F = 0.13). Figure 2 illustrates the mean proprioception scores at the three tested speeds.

### Effect of the 11+ Referees on dynamic balance of female futsal referees

**Fig. 3:**
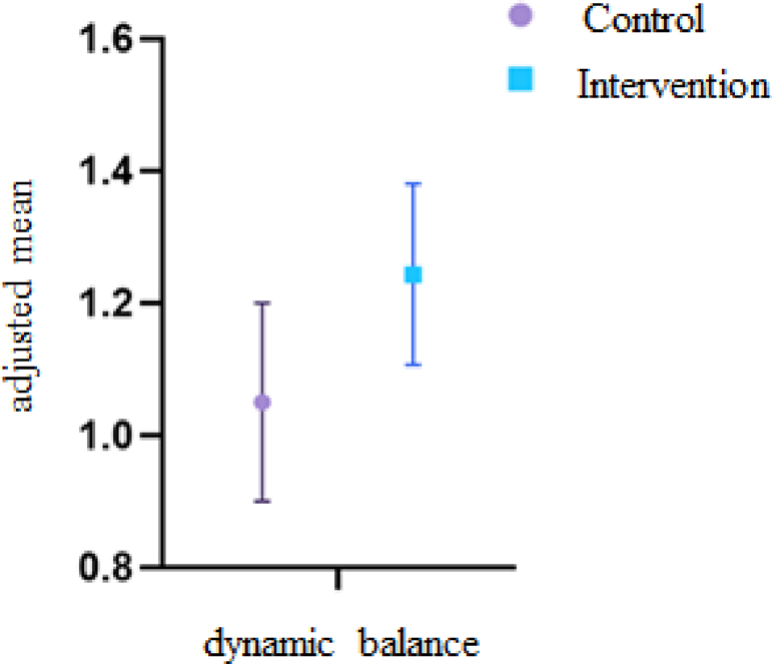
An adjusted mean difference of dynamic balance.

The ANCOVA results for dynamic balance revealed no significant difference between the control and intervention groups when controlling for pre-test values (ηp² = 0.033, *p* = 0.38, F = 0.79). Figure 3 presents the mean ± standard error for both groups.

## Discussion

This study examined the effects of an eight-week application of the 11 + Referees program on knee joint proprioception and dynamic balance in female futsal referees. The findings indicate that performing the 11 + Referees twice a week significantly improved knee joint proprioception compared to traditional warm-up routines but had no impact on dynamic balance and motion sense. Such lack of efficacy may be due to the short intervention period, lack of balance-specific exercise in the program, and the unique intermittent demands of indoor futsal refereeing, which might require more targeted balance drills.

To the best of the authors’ knowledge, only one other study has evaluated the effects of the 11 + Referees program on injury risk factors. By recruiting 81 elite referees, Alimoradi et al. (2024), investigated the impact of the 11 + Referees on change-of-direction quality using a cutting movement assessment score. Their results demonstrated significant improvements across all tested angles following the intervention^31^. While the limited body of literature makes it difficult to draw definitive conclusions, the available evidence indicates that the 11 + Referees program could enhance factors associated with knee injuries, such as proprioception and proper knee alignment. Given the higher risk of severe knee injuries among female referees compared to their male counterparts, integrating the 11 + Referees into their routine training could be beneficial, though further research is necessary to validate these findings and explore the program’s broader benefits.

Our findings align with those of Liu (2023), Seyedi et al. (2023) and Daneshjoo et al. (2012), supporting the idea that the FIFA11 + program can help develop knee proprioception. Liu et al. (2023), found that the FIFA + 11 program can effectively improve knee proprioception in young soccer players and may contribute to injury risk reduction in soccer players^32^. Similarly, Seyedi et al. (2023) observed notable improvements in joint position sense following application of the FIFA + 11 program among male and female adolescent soccer players^33^. of Daneshjoo et al. (2012), also highlighted that the FIFA + 11 program enhances knee joint position sense in both sexes of soccer players^34^. In contrast, Lopes et al. (2019), who studied amateur futsal players, found that neither the short-term nor long-term application of the FIFA + 11 could develop knee position sense^35^.

Known as an essential factor for movement efficiency and stability, knee joint proprioception relies on afferent information from deep receptors located in the capsules, ligaments, and muscle spindles. These receptors contribute to joint stability, postural control, and overall motor function^36^. Research suggests that proprioceptive deficits, rather than ligament damage alone, are a primary cause of joint instability, as they delay the reflexive activation of stabilizing muscle groups. ^32^. Studies further emphasize that active and dynamic exercise modalities such as those included in the FIFA 11 + and the 11 + Referees can better enhance proprioceptive function in athletes^32^. The sensorimotor system has various components that contribute to maintaining balance and functional stability of the joint^37^. Poor neuromuscular function can lead to a lack of balance and functional stability of the joint and various musculoskeletal injuries as a result^38^. By incorporating balance components, plyometric drills, and strength exercises, these interventions may promote neuromuscular adaptations that improve the accuracy of afferent feedback to the central nervous system (CNS), leading to better control of knee movement and improved joint position sense^33^.

Despite its positive effects on proprioception, our findings revealed that an eight-week application of the 11 + Referees did not improve the dynamic balance in female futsal referees. No significant improvement was observed in motion sense, which might be due to the limited sensitivity of the measurement tool or the intervention’s primary focus on proprioception at specific angular velocities rather than motion sense precision. This contrasts with previous research demonstrating that similar interventions, such as the FIFA11 + and the 11 + kids significantly improve the balance abilities of football players has been well documented^23^^39^. For instance, Liu et al. (2023) showed a notable improvement in balance ability following application of the FIFA + 11 among youth campus players^32^. However, two studies conducted by Lopes et al. (2019 & 2020) failed to show that performing the 11 + for ten weeks improves balance and proprioception and that the improvements persist ten weeks after the intervention^35^. Therefore, it seems likely that the combination of short intervention duration, lack of progression in balance-specific content, measurement sensitivity limitations, and the unique stop-and-go demands of futsal refereeing collectively explain the lack of significant change in dynamic balance in this study. Future research should explore the interaction between playing surface, balance performance, and exercise-based injury prevention programs.

## Conclusion

The 11 + Referees program appears to be an effective intervention for improving knee joint proprioception at lower (15°/s) and higher (60°/s) angular velocities in female futsal referees, which may contribute to injury prevention. Although the program did not significantly enhance dynamic balance, possibly due to the indoor hard court surface and lack of balance-specific drills, it still serves as a valuable addition to warm-up routines, especially given the high risk of non-contact knee injuries among female officials. It is recommended that coaches and refereeing bodies consider implementing the 11 + Referees program as part of the standard pre-match warm-up to improve proprioception and potentially reduce injury risk. Further research is needed to explore its long-term effects on other biomechanical measures and to determine whether modifying surface conditions or adding balance-focused exercises could yield improvements in dynamic balance.

## Implication

One limitation of this study is the relatively small sample size, which may reduce the generalizability of the results to all female futsal referees. Hence, further studies with greater sample sizes are required to confirm our findings. Another limitation is that potential confounding factors, such as individual differences in physical fitness level, prior training experience, and refereeing workload during the season, were not controlled for, which might have influenced the observed effects. Furthermore, the absence of a long-term follow-up prevents assessment of the durability of the program’s benefits over time.

## Data Availability

The data are available from the corresponding author under ([m_zareei@sbu.ac.ir](mailto: m_zareei@sbu.ac.ir)) on reasonable request.
